# High‐Humidity‐Tolerant Chloride Solid‐State Electrolyte for All‐Solid‐State Lithium Batteries

**DOI:** 10.1002/advs.202305394

**Published:** 2024-02-02

**Authors:** Kai Wang, Zhenqi Gu, Haoxuan Liu, Lv Hu, Ying Wu, Jie Xu, Cheng Ma

**Affiliations:** ^1^ School of Materials & Energy Lanzhou University Lanzhou Gansu 730000 China; ^2^ Hefei National Research Center for Physical Sciences at the Microscale CAS Key Laboratory of Materials for Energy Conversion Department of Materials Science and Engineering University of Science and Technology of China Hefei Anhui 230026 China; ^3^ Institute for Superconducting and Electronic Materials Australian Institute for Innovative Materials University of Wollongong Wollongong New South Wales 2525 Australia; ^4^ College of Chemistry and Materials Engineering Wenzhou University Wenzhou Zhejiang 325035 China; ^5^ National Synchrotron Radiation Laboratory Hefei Anhui 230026 China

**Keywords:** aliovalent substitution, all‐solid‐state lithium batteries, chloride solid‐state electrolytes, humidity tolerance

## Abstract

Halide solid‐state electrolytes (SSEs) hold promise for the commercialization of all‐solid‐state lithium batteries (ASSLBs); however, the currently cost‐effective zirconium‐based chloride SSEs suffer from hygroscopic irreversibility, low ionic conductivity, and inadequate thermal stability. Herein, a novel indium‐doped zirconium‐based chloride is fabricated to satisfy the abovementioned requirements, achieving outstanding‐performance ASSLBs at room temperature. Compared to the conventional Li_2_ZrCl_6_ and Li_3_InCl_6_ SSEs, the hc‐Li_2+x_Zr_1‐x_In_x_Cl_6_ (0.3 ≤ x ≤ 1) possesses higher ionic conductivity (up to 1.4 mS cm^−1^), and thermal stability (350 °C). At the same time, the hc‐Li_2.8_Zr_0.2_In_0.8_Cl_6_ also shows obvious hygroscopic reversibility, where its recovery rate of the ionic conductivity is up to 82.5% after 24‐h exposure in the 5% relative humidity followed by heat treatment. Theoretical calculation and experimental results reveal that those advantages are derived from the lattice expansion and the formation of Li_3_InCl_6_ ·2H_2_O hydrates, which can effectively reduce the migration energy barrier of Li ions and offer reversible hydration/dehydration pathway. Finally, an ASSLB, assembled with reheated‐Li_2.8_Zr_0.2_In_0.8_Cl_6_ after humidity exposure, single‐crystal LiNi_0.8_Mn_0.1_Co_0.1_O_2_ and Li‐In alloy, exhibits capacity retention of 71% after 500 cycles under 1 C at 25 °C. This novel high‐humidity‐tolerant chloride electrolyte is expected to greatly carry forward the ASSLBs industrialization.

## Graphical abstract

Herein, the humidity tolerance of halide solid‐state electrolytes using the soft acid element In‐doped Li_2_ZrCl_6_ is investigated and found that hc‐Li_0.8_Zr_0.2_In_0.8_Cl_6_ has high ionic conductivity, good thermal stability, and hygroscopic reversibility, which will greatly enhance the commercialization of all‐solid‐state lithium batteries.

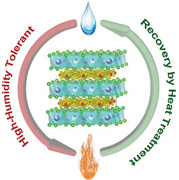

## 1 Introduction

The forthcoming era of next‐generation intelligent electronic devices and long‐range electric vehicles urges the development of advanced all‐solid‐state lithium batteries (ASSLBs) systems with higher safety, energy density, and longer lifespan.^[^ ^1^, ^2^, ^3^ ^]^ Replacing the liquid electrolytes with inorganic solid‐state electrolytes (SSEs) could not only offer solutions to the safety risks associated with liquid electrolytes, such as combustion and explosion but also promise improved energy density.^[^ ^4^, ^5^, ^6^ ^]^ Among inorganic SSEs, halide SSEs are particularly promising for commercial ASSLBs due to their high room‐temperature ionic conductivity, facile deformation, compatibility with oxide cathodes, low cost, and even a water‐based synthesis route.^[^ ^7^, ^8^, ^9^, ^10^ ^]^

To enable the large‐scale commercialization of halide SSEs, it is imperative to simultaneously enhance their hygroscopic irreversibility, ionic conductivity, and thermal stability. Currently, zirconium‐based chloride (Li_2_ZrCl_6_) is one of the most promising SSE materials, as it retains desirable properties of Li_3_MCl_6_ (M = Sc,^[^ ^11^ ^]^ Y,^[^ ^12^ ^]^ Tb−Lu,^[^ ^13^ ^]^ and In^[^ ^14^, ^15^ ^]^) systems, and exhibits a unique humidity tolerance at 5% relative humidity and low raw material cost.^[^ ^16^ ^]^ However, the Li_2_ZrCl_6_ SSE also suffers from hygroscopic irreversibility (>5% relative humidity), low ionic conductivity (<1 mS cm^−1^), and limited thermal stability. The hygroscopic irreversibility of zirconium‐based chloride could result in the increased cost of the synthesis, transportation, storage, and post‐processing, which is a major obstacle to the large‐scale production of halide SSEs. Although, the improvement in ionic conductivity and thermal stability for the zirconium‐based chloride could be achieved by adjusting the crystal structure from aperiodic to periodic via doping Sc,^[^ ^17^, ^18^ ^]^ Y,^[^ ^19^ ^]^ Yb,^[^ ^20^ ^]^ Er,^[^ ^21^ ^]^ Fe,^[^ ^22^ ^]^ V,^[^ ^22^ ^]^ Cr,^[^ ^22^ ^]^ Mg^[^ ^23^ ^]^ and In^[^ ^24^, ^25^ ^]^ into chlorides, the hygroscopic reversibility of zirconium‐based chlorides, which is crucial for industrialization, has not been improved until now. According to the Hard Soft Acid Base (HSAB) theory,^[^ ^26^, ^27^ ^]^ compounds formed from soft acids, such as In, Sn, and As (Li_3_InCl_6_,^[^ ^14^ ^]^ Li_4_SnS_4_^[^ ^28^ ^]^ and Li_3.833_Sn_0.833_As_0.166_S_4_^[^ ^28^ ^]^), are unlikely to undergo chemical reactions with the hard base H_2_O. Furthermore, Zhu et al. performed thermodynamic analyses based on first‐principles calculation databases to screen various halides for moisture‐stable elements like In, Cd, Zn, Ga, etc.^[^ ^29^ ^]^ The compounds based on the above elements exhibit hygroscopic reversibility, meaning they can be dehumidified and restored to their original state through heat treatment following exposure to moisture.^[^ ^30^ ^]^ Therefore, doping the soft acid elements into zirconium‐based chlorides is expected to improve their hygroscopic irreversibility, and enhance their room‐temperature ionic conductivity and thermal stability at the same time.

Herein, a novel SSE material, Li_2+x_Zr_1‐x_In_x_Cl_6_ (0 ≤ x ≤ 1), has been synthesized by doping with soft acid In‐ions, to simultaneously improve the hygroscopic irreversibility, ionic conductivity, and thermal stability. In detail, within the same crystal structure (*C2/m*), the changes from an aperiodic structure (Li_2_ZrCl_6_) to a periodic structure derived from doping In into the halide SSEs (Li_2+x_Zr_1‐x_In_x_Cl_6_ (0.2 ≤ x ≤ 1)) could result in better ionic conductivity (>1 mS cm^−1^) and thermal stability. Additionally, the In dopant enables the reversible hygroscopicity of Li_2+x_Zr_1‐x_In_x_Cl_6_ (0.8 ≤ x ≤ 1), which can be recovered from hygroscopicity by heat treatment. Based on those advantages, when applied in (ASSLBs) as the SSE and coupled with single‐crystal LiNi_0.8_Mn_0.1_Co_0.1_O_2_ (scNMC811) cathode and Li‐In alloy anode, hc‐Li_2.8_Zr_0.2_In_0.8_Cl_6_ and reheated‐Li_2.8_Zr_0.2_In_0.8_Cl_6_ after humidity exposure enables the ASSLBs to reach the first‐cycle Coulombic efficiency of 87.5% and 85.3% and discharge specific capacity of 174.5 and 169 mAh g^−1^ at 0.2 C, respectively. Furthermore, those ASSLBs display stable cycling performance, with 500 stable cycles at 1 C, accompanied by 74% and 71% capacity retention, respectively. This work offers a novel and facile strategy for realizing high hygroscopic reversibility, high‐safety, and high‐performance ASSLB, paving the way for its commercialization.

## 2 Results and Discussion

**Figure 1:**
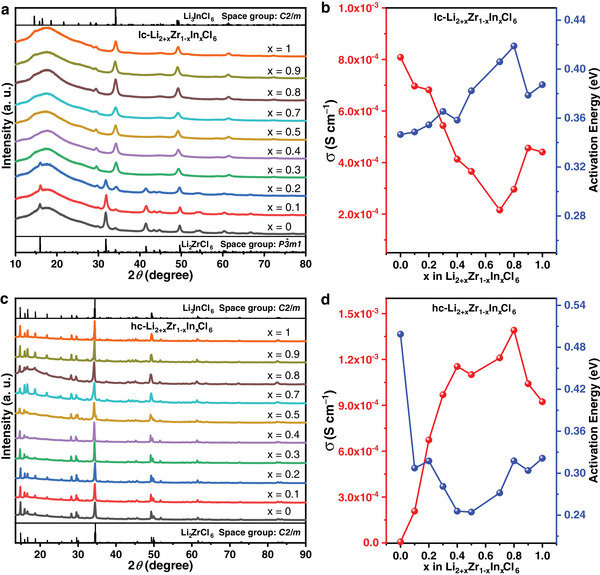
Crystal structures and ionic conductivities of Li_2+x_Zr_1‐x_In_x_Cl_6_ (0 ≤ x ≤ 1) under different processing conditions. a,c) XRD patterns of lc‐ and hc‐Li_2+x_Zr_1‐x_In_x_Cl_6_ (0 ≤ x ≤ 1). The Bragg positions of Li_3_InCl_6_ and Li_2_ZrCl_6_ are from references 15 and 16, respectively. b,d) The ionic conductivities (red dotted lines) and the activation energies (blue dotted lines) of lc‐ and hc‐Li_2+x_Zr_1‐x_In_x_Cl_6_ (0 ≤ x ≤ 1).

Li_2+x_Zr_1‐x_In_x_Cl_6_ (0 ≤ x ≤ 1) SSE materials were mechanochemically synthesized by a stoichiometric mixture of ZrCl_4_, InCl_3,_ and LiCl. The crystal structure of the materials was analyzed by X‐ray diffraction (XRD). As indicated in **Figure** **1**a, the diffraction peaks of the as‐milled Li_2+x_Zr_1‐x_In_x_Cl_6_ (0 ≤ x ≤ 1) SSE are almost entirely located at the main peak with low peak intensity, which is consistent with the law of low crystallinity of the high‐energy ball‐milled synthesized material.^[^ ^12^, ^16^, ^31^ ^]^ The low crystallinity material is referred to as lc‐Li_2+x_Zr_1‐x_In_x_Cl_6_ (0 ≤ x ≤ 1) below. The crystal structure of lc‐Li_2.1_Zr_0.9_In_0.1_Cl_6_ is similar to that of the as‐milled Li_2_ZrCl_6_ (trigonal with space group *P‐3m1*)^[^ ^16^ ^]^; the crystal structure of the lc‐Li_2+x_Zr_1‐x_In_x_Cl_6_ (0.3 ≤ x ≤ 0.9) is similar to the Li_3_InCl_6_ counterpart (monoclinic with space group *C2/m*)^[^ ^15^ ^]^; the diffraction peak of lc‐Li_2.2_Zr_0.8_In_0.2_Cl_6_ contains the structural features of *P‐3m1* and *C2/m*; thus, it may be a mixed phase. The In (80 pm, six‐coordinated^[^ ^23^ ^]^) and Zr (72 pm, six‐coordinated^[^ ^23^ ^]^) ions with similar ionic radii could form a continuous solid solution in the lc‐Li_2+x_Zr_1‐x_In_x_Cl_6_ (x = 0.1, 0.3 ≤ x ≤ 0.9) lattice, and the Zr ions with smaller ionic radii are more easily solid soluble in the Li_3_InCl_6_ lattice (*C2/m*). To determine the ionic and electronic conductivities of the lc‐Li_2+x_Zr_1‐x_In_x_Cl_6_ (0 ≤ x ≤ 1) SSE at room temperature, electrochemical impedance spectroscopy (EIS) and Hebb‐Wagner polarization measurements were performed, and the results are shown in Figure 1b, Figures S1, S2 (Supporting Information). In general, the room‐temperature ionic conductivity of lc‐Li_2+x_Zr_1‐x_In_x_Cl_6_ (0 ≤ x ≤ 1) is larger than 2.16 × 10^−4^ S cm^−1^ (Table S1, Supporting Information). Moreover, with increasing In content, the ionic conductivity of lc‐Li_2+x_Zr_1‐x_In_x_Cl_6_ (0 ≤ x ≤ 0.7) gradually decreases from 8.01 × 10^−4^ S cm^−1^ (lc‐Li_2_ZrCl_6_) to 2.16 × 10^−4^ S cm^−1^ (lc‐Li_2.7_Zr_0.3_In_0.7_Cl_6_), while the ionic conductivity of lc‐Li_2+x_Zr_1‐x_In_x_Cl_6_ (0.7 ≤ x ≤ 0.9) increases to 4.56 × 10^−4^ S cm^−1^ (lc‐Li_2.9_Zr_0.1_In_0.9_Cl_6_) with the In content rise. In particular, the ionic conductivities of lc‐Li_2_ZrCl_6_ and lc‐Li_3_InCl_6_ materials are 8.01 × 10^−4^ S cm^−1^ and 4.41 × 10^−4^ S cm^−1^, respectively. Furthermore, the activation energy of the lc‐Li_2+x_Zr_1‐x_In_x_Cl_6_ (0 ≤ x ≤ 1) have been calculated as shown in Figure 1b and Figure S1 (Supporting Information), which are comparable to that previously reported for Li_2_ZrCl_6_^[^ ^16^ ^]^ and Li_3_InCl_6_.^[^ ^15^ ^]^ Besides, the electronic conductivity of the lc‐Li_2+x_Zr_1‐x_In_x_Cl_6_ (0 ≤ x ≤ 1) material is between 3.60 × 10^−9^ S cm^−1^ and 8.02 × 10^−8^ S cm^−1^ (Figure S2 and Table S1, Supporting Information), which is at least four orders of magnitude less than the ionic conductivity, indicating that the lc‐Li_2+x_Zr_1‐x_In_x_Cl_6_ (0 ≤ x ≤ 1) are all pure ionic conductors.

Although the room‐temperature ionic conductivity of the lc‐Li_2+x_Zr_1‐x_In_x_Cl_6_ (0 ≤ x ≤ 1) is less than 1 mS cm^−1^, numerous studies have shown that heat treatment can improve the room‐temperature ionic conductivity of *C2/m*‐structured halide SSEs.^[^ ^11^, ^15^, ^30^ ^]^ To verify whether In dopant could improve the room‐temperature ionic conductivity of lc‐Li_2+x_Zr_1‐x_In_x_Cl_6_ (0.3 ≤ x ≤ 1), the material was vacuum‐sealed in a quartz tube and annealed at 350 °C for 5 h to improve its crystallinity. The XRD results show that compared to the lc‐Li_2+x_Zr_1‐x_In_x_Cl_6_ (0 ≤ x ≤ 1) material, the 350 °C‐annealed Li_2+x_Zr_1‐x_In_x_Cl_6_ (0 ≤ x ≤ 1) (hc‐Li_2+x_Zr_1‐x_In_x_Cl_6_ (0 ≤ x ≤ 1) has stronger diffraction peaks and correspondingly more Bragg peak positions, indicating their improved crystallinity (Figure 1c). Rietveld refinement shows that the diffraction peaks of the hc‐Li_2+x_Zr_1‐x_In_x_Cl_6_ (0 ≤ x ≤ 1) match well with the structural features of the *C2/m* space group, demonstrating the formation of a single‐phase continuum solid solution after doping with In (Figure S3, Supporting Information). Notably, the Rietveld refinement^[^ ^32^, ^33^ ^]^ uses the Li_3_InCl_6_ structures as the starting structure, with part of In atoms replaced by Zr at the same position. Tables S2−S11 (Supporting Information) show the lattice parameters and atomic coordinate information obtained from the Rietveld refinement of each component of the hc‐Li_2+x_Zr_1‐x_In_x_Cl_6_ (0 ≤ x ≤ 1). The unit‐cell volume of the hc‐Li_2+x_Zr_1‐x_In_x_Cl_6_ (0 ≤ x ≤ 0.8) increases as the In ions with larger ionic radii replace Zr ions with smaller ones; the unit‐cell volume reaches a maximum of 427.7 Å^3^ when x = 0.8 and the unit‐cell volume abnormally decreases again when x > 0.8. It has been suggested that the reduction in unit‐cell volume may be attributed to a decrease in Li vacancies and the simultaneous occupancy of In/Zr/Li atoms, as investigated by Helm et al.^[^ ^25^ ^]^ However, further confirmation of this hypothesis may require future studies employing spherical aberration‐corrected electron microscopy with enhanced spatial resolution.^[^ ^34^, ^35^ ^]^ Additionally, our findings demonstrate that as the In content increases, the hc‐Li_2+x_Zr_1‐x_In_x_Cl_6_ (0 ≤ x ≤ 1) expands predominantly along the *c*‐axis, while the expansion of the *a*‐ and *b*‐axes is comparatively limited, with a gradual decrease in the *β*‐angle (Figure S4, Supporting Information). These observations align with the conclusions drawn by Kwak et al.^[^ ^17^ ^]^ and Helm et al.^[^ ^25^ ^]^ The ionic conductivity of hc‐Li_2+x_Zr_1‐x_In_x_Cl_6_ (0 ≤ x ≤ 1) is close to or even exceeds 1 mS cm^−1^ in a wide range of 0.3 ≤ x ≤ 1, as shown in Figure 1d and Figure S5 (Supporting Information). In particular, the changes in their ionic conductivity are consistent with that of the unit‐cell volume, indicating that the increased ionic conductivity could be achieved by doping ions with a larger ionic radii. For hc‐Li_2+x_Zr_1‐x_In_x_Cl_6_ with compositions of x = 0.1 and x = 0.2, although their ionic conductivity does not increase significantly after annealing, they do not undergo significant degradation like hc‐Li_2_ZrCl_6_.^[^ ^16^ ^]^ Comparatively, the room‐temperature electronic conductivity of the hc‐ and lc‐Li_2+x_Zr_1‐x_In_x_Cl_6_ (0 ≤ x ≤ 1) does not change significantly and remains on the order of 10^−8^ S cm^−1^, indicating that all materials, except hc‐Li_2_ZrCl_6_, are pure ionic conductors (Figures S2, S6 and Tables S1, S12, Supporting Information). Compared to lc‐Li_2+x_Zr_1‐x_In_x_Cl_6_ (0 ≤ x ≤ 1), the hc‐Li_2+x_Zr_1‐x_In_x_Cl_6_ (0 ≤ x ≤ 1) has a lower activation energy after annealing, demonstrating a lower temperature dependence, which is favorable for the electrolyte materials to be applied in a wider temperature range (Figure 1b,d). Similar to the reported results,^[^ ^16^ ^]^ heat treatment is detrimental to the ionic conductivity of the Li_2_ZrCl_6_ material, with values decreasing from 8.08 × 10^−4^ to 6.11 × 10^−6^ S cm^−1^; whereas heat treatment is favorable for the ionic conductivity of the Li_3_InCl_6_ material, with values increasing from 4.41 × 10^−4^ to 9.23 × 10^−4^ S cm^−1^ (Table S12, Supporting Information). The ionic conductivity of hc‐Li_2.8_Zr_0.2_In_0.8_Cl_6_ reaches a maximum value of 1.4 × 10^−3^ S cm^−1^ (Table S12, Supporting Information). Notably, the ionic conductivity of hc‐Li_2+x_Zr_1‐x_In_x_Cl_6_ (0.3 ≤ x ≤ 1) is one order of magnitude higher than the lc‐Li_2+x_Zr_1‐x_In_x_Cl_6_ (0.3 ≤ x ≤ 1) counterpart, indicating its better thermal stability.

**Figure 2:**
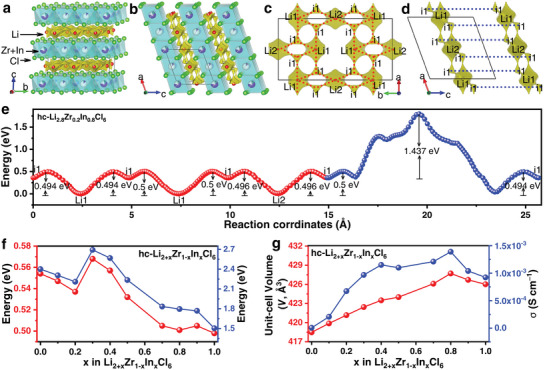
BVSE analysis of Li‐ion migration within hc‐Li_2+x_Zr_1‐x_In_x_Cl_6_ (0 ≤ x ≤ 1). a,b) The crystal structure models of hc‐Li_2.8_Zr_0.2_In_0.8_Cl_6_ superimposed with the Li‐ion potential map. c−e) Li‐ion migration pathways (c,d) and associated energy profiles (e) of hc‐Li_2.8_Zr_0.2_In_0.8_Cl_6_. f) Li‐ion migration energy profiles of hc‐Li_2+x_Zr_1‐x_In_x_Cl_6_ (0 ≤ x ≤ 1). g) The unit‐cell volume (red dotted line) and ionic conductivities (blue dotted line) of hc‐Li_2+x_Zr_1‐x_In_x_Cl_6_ (0 ≤ x ≤ 1).

To investigate the mechanism of the ionic conductivity of hc‐Li_2+x_Zr_1‐x_In_x_Cl_6_ (0 ≤ x ≤ 1), the bond valence site energy (BVSE) analysis^[^ ^36^, ^37^ ^]^ was performed using the results from the Rietveld refinement. As the crystal structures of hc‐Li_2+x_Zr_1‐x_In_x_Cl_6_ (0 ≤ x ≤ 1) SSEs are all consistent (**Figure** **2**a,b), the hc‐Li_2.8_Zr_0.2_In_0.8_Cl_6_ with the highest ionic conductivity was selected as an example to determine its Li‐ion migration paths and energy barriers, as shown in Figure 2c−e. For hc‐Li_2.8_Zr_0.2_In_0.8_Cl_6_, the red migration path within the a‐b plane (Figure 2c,d) is relatively favorable for Li‐ion migration and exhibits an effective energy barrier of 0.5 eV (Figure 2e). In contrast, the blue migration path between two adjacent a‐b planes (Figure 2c,d) has a higher migration energy barrier (1.796 eV). It should be emphasized that such a result cannot be stated with certainty because Li‐ions migrate in two dimensions, and the Zr/In sites that hinder the Li‐ion migration between the two a‐b planes are not fully occupied^[^ ^16^, ^31^ ^]^ (Table S9, Supporting Information). As the hc‐Li_2+x_Zr_1‐x_In_x_Cl_6_ (0 ≤ x ≤ 1) is a layered structure and the Li‐ions migrate within the a‐b plane against less resistance than that in the neighboring a‐b plane, the effective migration energy barrier for the hc‐Li_2+x_Zr_1‐x_In_x_Cl_6_ (0 ≤ x ≤ 1) can be simplified to that of the a‐b plane. As indicated in Figure 2f, the effective migration energy barrier for the hc‐Li_2+x_Zr_1‐x_In_x_Cl_6_ (0 ≤ x ≤ 1) is calculated by BVSE. All migration energy barriers of the Li‐ion migration paths are small when x < 0.3, but the corresponding ionic conductivity is not high. Combining with XRD and EIS results, we speculate that the ionic conductivity of hc‐Li_2+x_Zr_1‐x_In_x_Cl_6_ (0 ≤ x ≤ 1) mainly depends on the aperiodic structure. All migration energy barriers of the Li‐ion migration paths gradually decrease with the increase of In content when 0.3 ≤ x ≤ 0.8, due to the lattice expansion in the *a*‐, *b*‐, and *c*‐directions, and expansion of the unit‐cell volume caused by In doping (Figure 2g). And the gradual increase in their ionic conductivity is also consistent with the change in the migration energy barrier. The anomalous contraction of the lattice leads to an increase in the migration energy barrier of its Li‐ion migration path when x = 0.9, which in turn leads to a decrease in ionic conductivity. In addition, Li_3_InCl_6_ seems to have a slightly lower ionic conductivity, probably because the complete disappearance of Li vacancies hinders the migration of Li‐ions.

**Figure 3:**
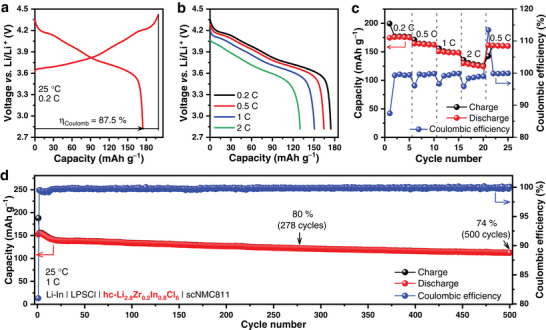
Electrochemical performance of the Li‐In│LPSCl│hc‐Li_2.8_Zr_0.2_In_0.8_Cl_6_│scNMC811 cell. a) The initial charge/discharge curves under 0.2 C at 25 °C. b,c) Rate capability under 0.2 C, 0.5 C, 1 C, and 2 C at 25 °C. d) Long‐term cycling performance under 1 C at 25 °C.

Based on the above discussion, a series of In‐doped hc‐Li_2+x_Zr_1‐x_In_x_Cl_6_ SSEs have been confirmed to possess good thermal stability and room‐temperature ionic conductivity approaching or even exceeding 1 mS cm^−1^ over a wide range of 0.3 ≤ x ≤ 1. To further investigate their electrochemical performance, the hc‐Li_2.8_Zr_0.2_In_0.8_Cl_6_ with highest ionic conductivity was selected as a representative SSE to assemble ASSLBs, with scNMC811 as the cathode and Li‐In alloy as the anode. As both Li_2_ZrCl_6_ and Li_3_InCl_6_ are unstable with the Li‐In alloy, a thin layer of Li_6_PS_5_Cl (LPSCl) was added between the hc‐Li_2.8_Zr_0.2_In_0.8_Cl_6_ SSE and the Li‐In alloy to prevent side reactions during the assembly process of ASSLBs. The Li‐In│LPSCl│hc‐Li_2.8_Zr_0.2_In_0.8_Cl_6_│scNMC811 cell cycled between 2.82−4.42 V versus Li/Li^+^ at 0.2 C (1 C = 200 mA g^−1^), showing an initial Coulombic efficiency of up to 87.5% and a discharge specific capacity of 174.5 mAh g^−1^ (**Figure** **3**a). The specific capacity of the cell decreased only slightly when the rate increased from 0.2 C to 0.5 C; the average discharge specific capacity was 175.5, 163.3, and 149.2 mAh g^−1^ at 0.2 C, 0.5 C, and 1 C, respectively (Figure 3b,c). Even at 2 C, an average capacity of 127.0 mAh g^–1^ was still retained, indicating that the cell has good rate performance. The Li‐In│LPSCl│hc‐Li_2.8_Zr_0.2_In_0.8_Cl_6_│scNMC811 cell maintained 80% and 74% capacity retention respectively after 278 and 500 cycles under 1 C at 25 °C (Figure 3d). It is noteworthy that cells assembled with hc‐Li_2.8_Zr_0.2_In_0.8_Cl_6_ also have similar electrochemical properties compared to cells assembled with Li_2_ZrCl_6_^[^ ^16^ ^]^ and Li_3_InCl_6_^[^ ^15^ ^]^ as SSEs. Excessive introduction of In undoubtedly increases the cost of raw materials, which is not favorable for large‐scale applications. Therefore, we further investigated the electrochemical performance at an In content of 0.3 (Figure S7, Supporting Information), which showed comparable performance to that of In 0.8, such as 80% capacity retention after 325 cycles. Therefore, hc‐Li_2+x_Zr_1‐x_In_x_Cl_6_ (0.3 ≤ x ≤ 1) combines high ionic conductivity, thermal stability, and good electrochemical properties. The continuous tunability of the In content, while maintaining performance, provides a solution for the manufacture of low‐cost halide SSEs.

**Figure 4:**
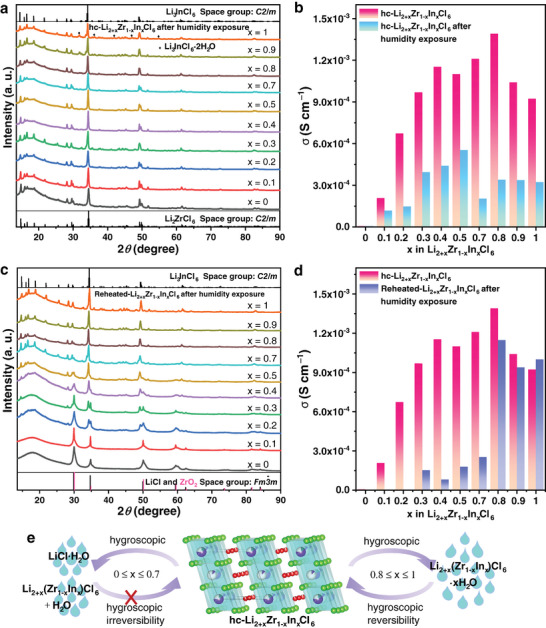
The humidity tolerance of hc‐Li_2+x_Zr_1‐x_In_x_Cl_6_ (0 ≤ x ≤ 1). a) XRD patterns of hc‐Li_2+x_Zr_1‐x_In_x_Cl_6_ (0 ≤ x ≤ 1) after 24 h of exposure in 5% relative humidity. b) The ionic conductivities of the hc‐Li_2+x_Zr_1‐x_In_x_Cl_6_ (0 ≤ x ≤ 1) before and after 24 h of exposure in 5% relative humidity. c) XRD patterns of the reheated‐Li_2+x_Zr_1‐x_In_x_Cl_6_ (0 ≤ x ≤ 1). d) The ionic conductivities of the hc‐Li_2+x_Zr_1‐x_In_x_Cl_6_ (0 ≤ x ≤ 1) and the reheated‐Li_2+x_Zr_1‐x_In_x_Cl_6_ (0 ≤ x ≤ 1). e) Schematic diagram of the humidity tolerance of the hc‐Li_2+x_Zr_1‐x_In_x_Cl_6_ (0 ≤ x ≤ 1).

Apart from ionic conductivity and thermal stability, the hygroscopic irreversibility is the most important factor limiting the large‐scale production, transportation, and storage of zirconium‐based chloride. The influence of partial doping of the soft acid element In on the humidity tolerance of hc‐Li_2+x_Zr_1‐x_In_x_Cl_6_ (0 ≤ x ≤ 1) deserves further investigation. Li_3_InCl_6_ synthesized from soft acidic elements does not react chemically with the hard base H_2_O but forms Li_3_InCl_6_⋅2H_2_O hydrates, which can be annealed to remove the crystalline water and restore the ionic conductivity of Li_3_InCl_6_.^[^ ^14^ ^]^ Therefore, the water‐based synthesis of Li_3_InCl_6_ is suitable for large‐scale production.^[^ ^14^ ^]^ To test the humidity tolerance of the hc‐Li_2+x_Zr_1‐x_In_x_Cl_6_ (0 ≤ x ≤ 1), it was exposed to 5% relative humidity for 24 h, after which its crystal structure and conductivity were checked as shown in **Figures** **4**a,b and S8 (Supporting Information). The XRD results show that the main diffraction peaks of hc‐Li_2+x_Zr_1‐x_In_x_Cl_6_ (0 ≤ x ≤ 0.7) after 24 h of exposure to 5% relative humidity are not significantly different from those before the exposure, indicating that its weak hygroscopicity does not destroy its main crystalline structure or that it is similar to the case of Li_2_ZrCl_6_ where the products formed after exposure to 5% relative humidity are amorphous. When x > 0.8, traces of the Li_3_InCl_6_⋅2H_2_O crystal phase can be detected in the XRD spectrum, where the diffraction peak of the Li_3_InCl_6_⋅2H_2_O crystal phase is most prominent at x = 1 (Figure 4a).^[^ ^14^, ^16^, ^30^ ^]^ The EIS results show that the ionic conductivity of hc‐Li_2+x_Zr_1‐x_In_x_Cl_6_ (0 ≤ x ≤ 1) SSEs decreases after exposure to 5% relative humidity, especially when 0.3 ≤ x ≤ 1, and the ionic conductivity decreases by almost one order of magnitude (Figure 4b; Figure S8, Supporting Information). However, the ionic conductivity of Li_2_ZrCl_6_ is comparable to that of the unexposed samples, but this result cannot confirm its humidity tolerance. This is because its ionic conductivity is too low and even a product after reaction with water may have a comparable ionic conductivity. Besides, there is no obvious difference in the electronic conductivities of the hc‐Li_2+x_Zr_1‐x_In_x_Cl_6_ (0 ≤ x ≤ 1) before and after 24 h of exposure to 5% relative humidity, where the values remain in the order of 10 ^−8^ S cm^−1^, indicating that the exposed hc‐Li_2+x_Zr_1‐x_In_x_Cl_6_ (0.1 ≤ x ≤ 1) is still an ionic conductor instead of an electronic conductor (Figure S8 and Table S13, Supporting Information). From the fact that the ionic conductivity drops to at least 50%, it is clear that hc‐Li_2+x_Zr_1‐x_In_x_Cl_6_ (0 ≤ x ≤ 1) does not appear to be humidity‐tolerant at 5% relative humidity.

However, according to the conclusion that Li_3_InCl_6_ has hygroscopic reversibility, its product Li_3_InCl_6_⋅2H_2_O can completely remove two water molecules at high temperatures.^[^ ^14^ ^]^ We speculate that hc‐Li_2+x_Zr_1‐x_In_x_Cl_6_ (0.8 ≤ x ≤ 1) may also hold similar hygroscopic reversibility. To verify this speculation, the hc‐Li_2+x_Zr_1‐x_In_x_Cl_6_ (0 ≤ x ≤ 1) after 24 h of exposure in 5% relative humidity was dehydrated in a vacuum desiccator and then reannealed at 350 °C for 5 h. The final product is hereafter referred to as reheated‐Li_2+x_Zr_1‐x_In_x_Cl_6_ (0 ≤ x ≤ 1)), and its crystal structure is analyzed. The XRD results show that the crystal structures of the reheated‐Li_2+x_Zr_1‐x_In_x_Cl_6_ (0 ≤ x ≤ 0.7) are totally changed, which match well with the LiCl and ZrO_2_, proving that the hydrated compounds formed after exposure in 5% relative humidity are amorphous (Figure 4c). In addition, the LiCl and ZrO_2_ contents decreased from 100% to 10.07% with the In content increasing from 0 to 0.7 (Figure S9 and Table S14, Supporting Information). In contrast, hc‐Li_2+x_Zr_1‐x_In_x_Cl_6_ (0.8 ≤ x ≤ 1), due to its hygroscopicity, produces a crystal phase of Li_3_InCl_6_⋅2H_2_O when exposed to a humid environment, where it could remove the crystal water and change back to the original material after being annealed at 350 °C. At this point, the decomposition impurities LiCl and ZrO_2_ were only 3.04% at x = 0.8. At x = 0.9, the percentage is even reduced to 0 (Figure S9 and Table S14, Supporting Information). It is noteworthy that the ZrCl_6_^2−^ unit is still present in both reheated‐Li_2.8_Zr_0.2_In_0.8_Cl_6_ and reheated‐Li_2.9_Zr_0.1_In_0.9_Cl_6_. The characteristic peaks of these samples exhibit a leftward shift when compared to reheated‐Li_3_InCl_6_ (Figure S10, Supporting Information), which aligns with the previously discussed reduction in unit‐cell volume. Furthermore, the lattice parameter of reheated‐Li_2+x_Zr_1‐x_In_x_Cl_6_ (0.8 ≤ x ≤ 1) remains nearly unchanged when compared to hc‐Li_2+x_Zr_1‐x_In_x_Cl_6_ (0.8 ≤ x ≤ 1) (Tables S15−S17, Supporting Information). The conductivity of the reheated‐Li_2+x_Zr_1‐x_In_x_Cl_6_ (0 ≤ x ≤ 1) is shown in Figure 4d, Figure S11, and Table S18 (Supporting Information). The ionic conductivity of the reheated‐Li_2+x_Zr_1‐x_In_x_Cl_6_ (0 ≤ x ≤ 0.7) is not recovered, since their crystal structures are severely disturbed; however, compared to the original samples, the recovery rates of the ionic conductivities of hc‐Li_2.8_Zr_0.2_In_0.8_Cl_6_, hc‐Li_2.9_Zr_0.1_In_0.9_Cl_6,_ and hc‐Li_3_InCl_6_ are 82.5%, 90.2%, and 108.3%, respectively. The electron conductivity does not change intrinsically, indicating that both the hygroscopic and decomposition products are electron insulators (Tables S12, S13, S18, Supporting Information). Therefore, when the content of In exceeds 0.8, the samples not only have high ionic conductivity and good thermal stability but more importantly, they show hygroscopic reversibility, which is essential for their large‐scale industrial application (Figure 4e).

**Figure 5:**
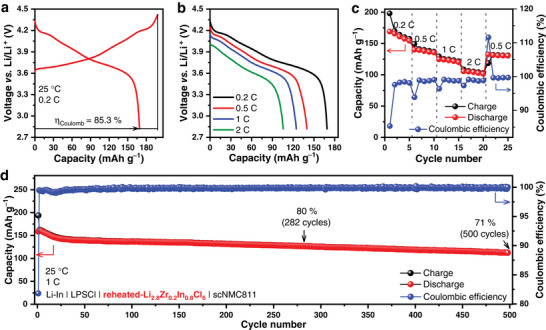
Electrochemical performance of the Li‐In│LPSCl│reheated‐Li_2.8_Zr_0.2_In_0.8_Cl_6_│scNMC811 cell. a) The initial charge/discharge curves under 0.2 C at 25 °C. b,c) Rate capability under 0.2 C, 0.5 C, 1 C, and 2 C at 25 °C. d) Long‐term cycling performance under 1 C at 25 °C.

To further investigate the effect of humidity exposure on the electrochemical performance of Li_2.8_Zr_0.2_In_0.8_Cl_6_, hc‐Li_2.8_Zr_0.2_In_0.8_Cl_6_ was replaced by reheated‐Li_2.8_Zr_0.2_In_0.8_Cl_6_ after humidity exposure in all‐solid‐state cell. The Li‐In│LPSCl│reheated‐Li_2.8_Zr_0.2_In_0.8_Cl_6_│scNMC811 cell cycled between 2.82−4.42 V versus Li/Li^+^ at 0.2 C (1 C = 200 mA g^−1^), showing an initial Coulombic efficiency of up to 85.3% and a discharge specific capacity of 169 mAh g^−1^ (**Figure** **5a**). It has similar rate performance to Li‐In│LPSCl│hc‐Li_2.8_Zr_0.2_In_0.8_Cl_6_│scNMC811 cell (Figures 3b,c and 5b,c) and maintained 80% and 71% capacity retention respectively after 282 and 500 cycles under 1 C at 25 °C (Figure 5d). Therefore, In doping improves the hygroscopic reversibility of zirconium‐based chlorides without affecting their electrochemical properties.

## 3 Conclusion

In summary, we report the Li_2+x_Zr_1‐x_In_x_Cl_6_ (0 ≤ x ≤ 1), a halide SSE with high room‐temperature ionic conductivity, good thermal stability, and hygroscopic reversibility. The room‐temperature ionic conductivity of the hc‐Li_2+x_Zr_1‐x_In_x_Cl_6_ (0.3 ≤ x ≤ 1) can reach 1 mS cm^−1^ after doping In. The key to high ionic conductivity for the SSE is the lattice expansion which can reduce the migration energy barrier for Li‐ions, and the annealing process is also beneficial to improving conductivity. In addition, the In dopant could enable the hc‐Li_2+x_Zr_1‐x_In_x_Cl_6_ (0.8 ≤ x ≤ 1) to possess hygroscopic reversibility, where after exposure to 5% relative humidity, its ionic conductivity can be restored by heat treatment to 82.5%, 90.2% and 108.3% at x = 0.8, 0.9 and 1, respectively. Furthermore, the ASSLBs assembled with hc‐Li_2.8_Zr_0.2_In_0.8_Cl_6_ and reheated‐Li_2.8_Zr_0.2_In_0.8_Cl_6_ after humidity exposure exhibit high first‐cycle Coulombic efficiency of 87.5% and 85.3%; discharge‐specific capacity of 174.5 and 169 mAh g^−1^ at 0.2 C, respectively. Furthermore, they can be stably cycled for 500 cycles, with capacity retention rates of 74% and 71% at 1 C, respectively. The outstanding performance of the Li_2+x_Zr_1‐x_In_x_Cl_6_ (0 ≤ x ≤ 1) SSE on the ionic conductivity, thermal stability, and humidity tolerance can promote its industrial application and provide novel ideas to optimize the overall performance of other halide SSEs.

## 4 Experimental Methods

### Materials Synthesis

Li_2+x_Zr_1‐x_In_x_Cl_6_ (0 ≤ x ≤ 1) SSEs were synthesized mechanochemically by mixing LiCl (Thermo Scientific, 99.9%), ZrCl_4_ (Thermo Scientific, 98%) and InCl_3_ (Thermo Scientific, 99.99%) in different stoichiometric ratios. The milling process was performed in a planetary mill of FRITSCH (PULVERISETTE 7 premium line) with a Si_3_N_4_ pot (80 mL) and ZrO_2_ balls (10, 5, and 3 mm in diameter) at 500 rpm for 45 h, and the ball‐to‐powder mass ratio was 10:1. Then, the materials were vacuum sealed in a quartz tube and annealed at 350 °C for 5 h. The filling into and removal from the quartz tube and the ball mill tank of the Li_2+x_Zr_1‐x_In_x_Cl_6_ (0 ≤ x ≤ 1) were carried out in a glove box (H_2_O < 0.01 ppm, O_2_ < 0.01 ppm) protected by Argon gas (Ar).

### Structural Characterization

X‐ray diffraction (XRD) was measured in a diffractometer (Rigaku Ultima IV) with Cu Kα1 radiation at room temperature, and the scanning speed was 5 ° min^−1^. The Li_2+x_Zr_1‐x_In_x_Cl_6_ (0 ≤ x ≤ 1) SSE powder was sealed in Kapton films to prevent exposure to humid air. The process was conducted within an Ar‐filled glovebox with H_2_O < 0.01 ppm and O_2_ < 0.01 ppm. Rietveld refinement was performed using the GSAS II program.^[^ ^32^, ^33^ ^]^ The overall atomic ratios of Li, Zr, In, and Cl were kept proportional during the refinement. The bond valence site energy (BVSE) analysis was performed using the softBV program,^[^ ^36^, ^37^ ^]^ and the structural models of the hc‐Li_2+x_Zr_1‐x_In_x_Cl_6_ (0 ≤ x ≤ 1) were obtained from Rietveld refinement.

### Conductivity Measurements

The ionic conductivities were calculated by electrochemical impedance spectroscopy (EIS) measurement, which was performed using an MTZ‐35 impedance analyzer (Bio‐Logic) with 20 mV driving potential amplitude in the frequency range of 35 MHz to 1 Hz. The electronic conductivities were calculated by direct current (DC) polarization measurement, which was performed using an electrochemical workstation (CHI630E) from CH Instruments Inc. with an applied voltage of 1 V. The SSE pellets used for the measurement of ionic and electronic conductivities were obtained by cold pressing at 380 MPa in a mold with a diameter of 11 mm without heat treatment, and Au electrodes were sputtered on the pellet surfaces. Notably, there was no additional stress during the measurement.

### Humidity Tolerance Test

Humidity tolerance tests were performed by exposing 0.5 g of hc‐Li_2+x_Zr_1‐x_In_x_Cl_6_ (0 ≤ x ≤ 1) chloride SSE powder to a vacuum desiccator filled with nitrogen gas (N_2_) at 5% relative humidity for 24 h at 25 °C. The humid environment was a mixture of dry N_2_ and N_2_ containing H_2_O in a certain ratio.^[^ ^16^ ^]^ A humidity sensor has been placed in the vacuum desiccator to ensure that the provided humid environment has the desired humidity. After 24 h in a humid environment at 5% relative humidity, the samples were dried in a vacuum oven at 60, 80, 120, and 200 °C for 5 h to remove water from crystallization. Finally, the dried samples were vacuum‐sealed in quartz tubes and then heat‐treated at 350 °C for 5 h.

### Electrochemical Characterizations

Before assembling the ASSLBs, the cathode composite was prepared by mixing the hc‐Li_2.8_Zr_0.2_In_0.8_Cl_6_ or reheated‐Li_2.8_Zr_0.2_In_0.8_Cl_6_ with the single‐crystal LiNi_0.8_Mn_0.1_Co_0.1_O_2_ (scNMC811) powder (Hunan Shanshan Energy Technology, 99.9%) at a 25:75 weight ratio in an Ar‐filled glove box (H_2_O < 0.01 ppm, O_2_ < 0.01 ppm) using a vortex mixer (Haimen Kylin‐Bell Lab Instruments, QL‐866) for 10 min. To assemble the Li‐In│LPSCl│hc‐Li_2.8_Zr_0.2_In_0.8_Cl_6_│scNMC811 and Li‐In│LPSCl│reheated‐Li_2.8_Zr_0.2_In_0.8_Cl_6_│scNMC811 cells, 60 mg of hc‐Li_2.8_Zr_0.2_In_0.8_Cl_6_ or reheated‐Li_2.8_Zr_0.2_In_0.8_Cl_6_ was placed into a polyetheretherketone (PEEK) mold with an inner diameter of 10 mm and was compacted into pellets with a pressure of 0.6 tons. Then, 40 mg of Li_6_PS_5_Cl (LPSCl, Ganfeng Lithium Group Co., Ltd, 99%) powder was spread on the hc‐Li_2.8_Zr_0.2_In_0.8_Cl_6_ or reheated‐Li_2.8_Zr_0.2_In_0.8_Cl_6_ layer and pressed under 1 ton of pressure. After inverting the PEEK mold, we sprinkled 7 mg of the cathode composite evenly over the other side of the hc‐Li_2.8_Zr_0.2_In_0.8_Cl_6_ or reheated‐Li_2.8_Zr_0.2_In_0.8_Cl_6_ layer and pressed it under 3 tons of pressure. Finally, 0.1 mm thick indium foil with a diameter of 10 mm (Qinghe County Chengshuo Metal Materials Co., Ltd, 99.995%) and 0.05 mm thick lithium foil with a diameter of 10 mm (China Energy Lithium Co., Ltd, 99.9%) were placed on top of the LPSCl layer in the order of indium foil first and then lithium foil, and pressed under 1 ton of pressure. All cells were assembled in an Ar‐filled glove box (H_2_O < 0.01 ppm, O_2_ < 0.01 ppm). After being assembled, the cells were sealed and transferred to a 25 °C atmospheric environment incubator with temperature accuracy ±1 °C (Tianjin Hongnuo Instrument, SPX‐250B) for electrochemical testing under an external mold pressure of 1.5 tons.

## Conflict of Interest

The authors declare no conflict of interest.

## Author Contributions

K.W., Z.G., and H.L. contributed equally to this work. K.W., J.X., and C.M. conceived the research. With the assistance of Z.G. and L.H., K.W. performed the materials synthesis, conductivity measurement, structural characterization, Rietveld refinement and BVSE analysis. Z.G. performed the X‐ray diffraction experiments and electrochemical tests. With the assistance of Y.W., K.W. analyzed the experimental data and wrote the manuscript. H.L. revised the manuscript. K.W., J.X., and C.M. directed the entire study.

## Supporting information

Supporting Information

## Data Availability

The data that support the findings of this study are available in the supplementary material of this article.
