# Supplementary material for: High‐Humidity‐Tolerant Chloride Solid‐State Electrolyte for All‐Solid‐State Lithium Batteries
Source: Adv Sci (Weinh). 2024 Feb 2;11(14):2305394. doi: 10.1002/advs.202305394 (PMC11005720; doi:10.1002/advs.202305394)
Supplement: Supplementary file 1 — Supporting Information [file ADVS-11-2305394-s001.pdf]

ADVANCED SCIENCE
Open Access

Supporting Information

for *Adv. Sci.*, DOI 10.1002/advs.202305394

High-Humidity-Tolerant Chloride Solid-State Electrolyte for All-Solid-State Lithium Batteries

Kai Wang, Zhenqi Gu, Haoxuan Liu, Lv Hu, Ying Wu, Jie Xu* and Cheng Ma**

Supporting Information

High-Humidity-Tolerant Chloride Solid-State Electrolyte for All-Solid-State Lithium Batteries

Kai Wang[+], Zhenqi Gu[+], Haoxuan Liu[+], Lv Hu, Ying Wu, Jie Xu* and Cheng Ma**

K. Wang, Y. Wu
School of Materials & Energy
Lanzhou University
Lanzhou, Gansu 730000, China
E-mail: kwang@lzu.edu.cn

K. Wang, Z. Gu, L. Hu, C. Ma
Hefei National Research Center for Physical Sciences at the Microscale
CAS Key Laboratory of Materials for Energy Conversion
Department of Materials Science and Engineering
University of Science and Technology of China
Hefei, Anhui 230026, China
E-mail: mach16@ustc.edu.cn

H. Liu
Institute for Superconducting and Electronic Materials
Australian Institute for Innovative Materials, University of Wollongong
Wollongong, New South Wales 2525, Australia

J. Xu
College of Chemistry and Materials Engineering
Wenzhou University
Wenzhou, Zhejiang 325035, China

E-mail: jiexu@wzu.edu.cn

C. Ma

National Synchrotron Radiation Laboratory

Hefei, Anhui 230026, China

+The authors contributed equally to this work.

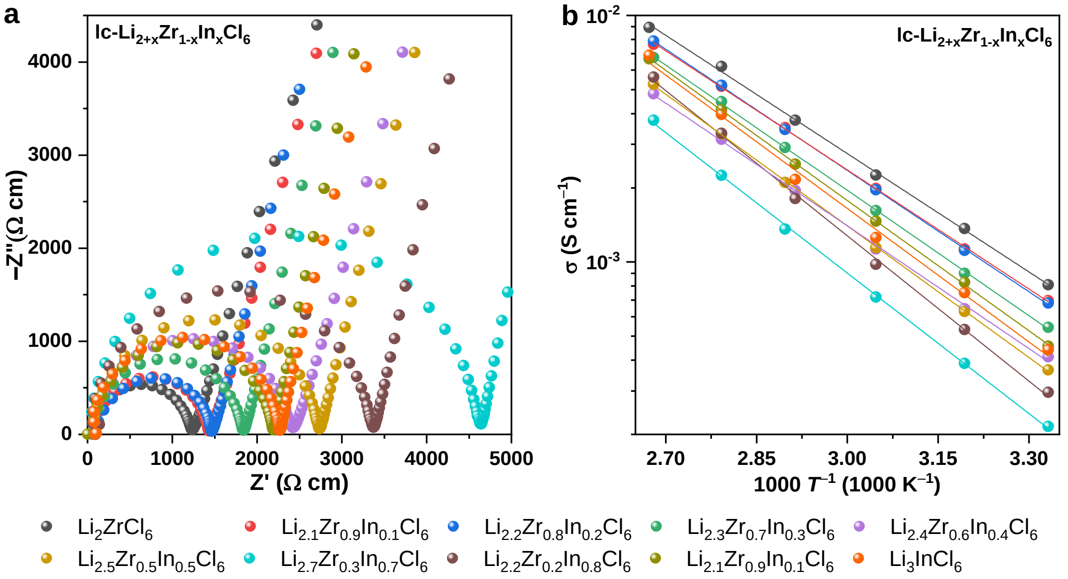

Figure S1. Nyquist (a) and Arrhenius (b) plots of the lc-$Li_{2+x}Zr_{1-x}In_xCl_6$ ($0 \leq x \leq 1$) materials.

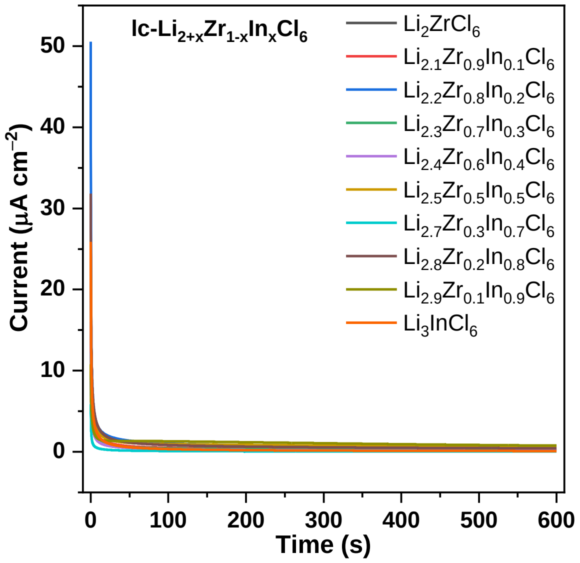

Figure S2. The transient current behaviour under an applied 1.0 V DC bias on the lc-$Li_{2+x}Zr_{1-x}In_xCl_6$ ($0 \leq x \leq 1$) pellets with stainless-steel electrodes.

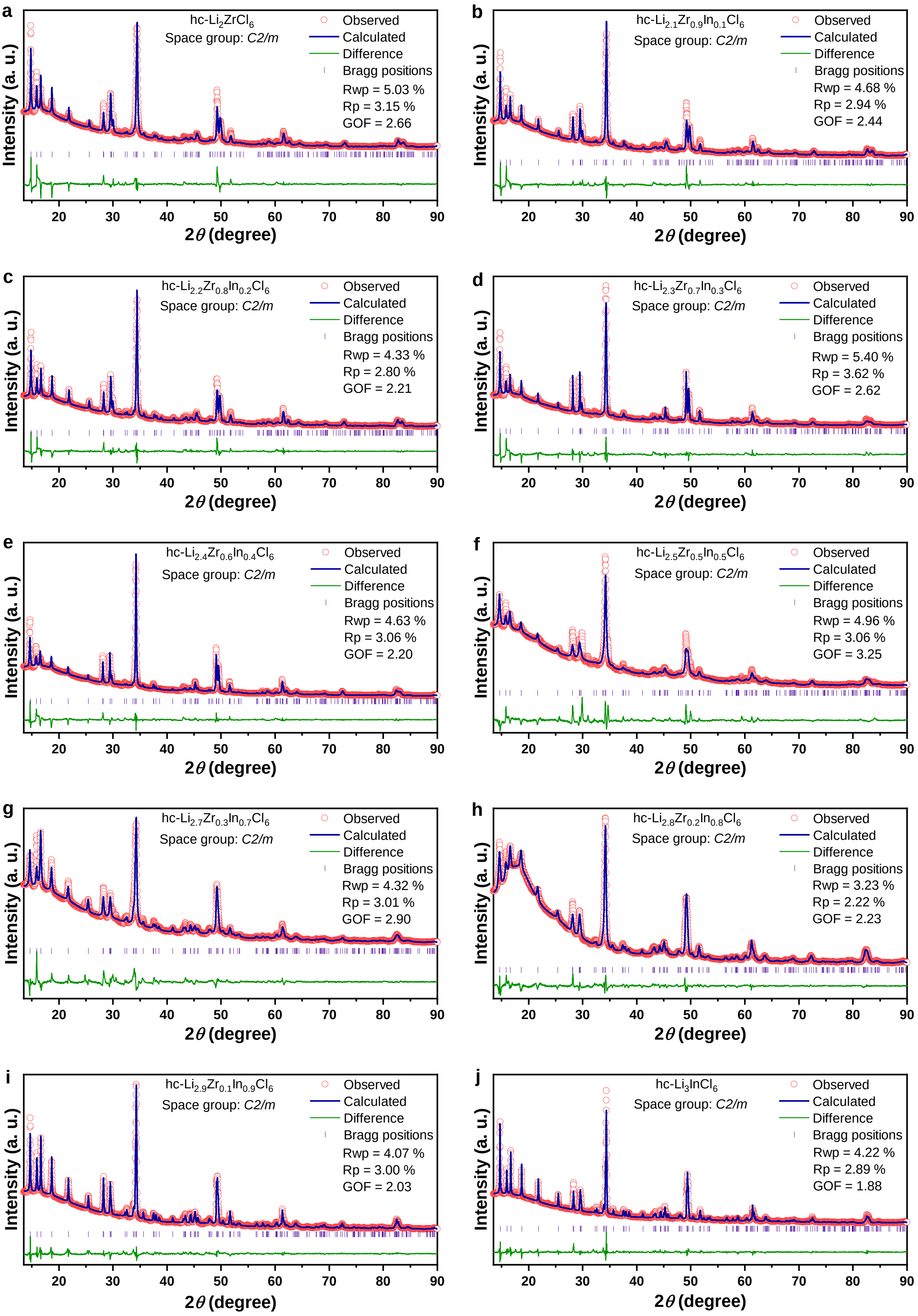

Figure S3. (a–j) Refined XRD patterns of the hc-$Li_{2+x}Zr_{1-x}In_xCl_6$ ($0 \leq x \leq 1$).

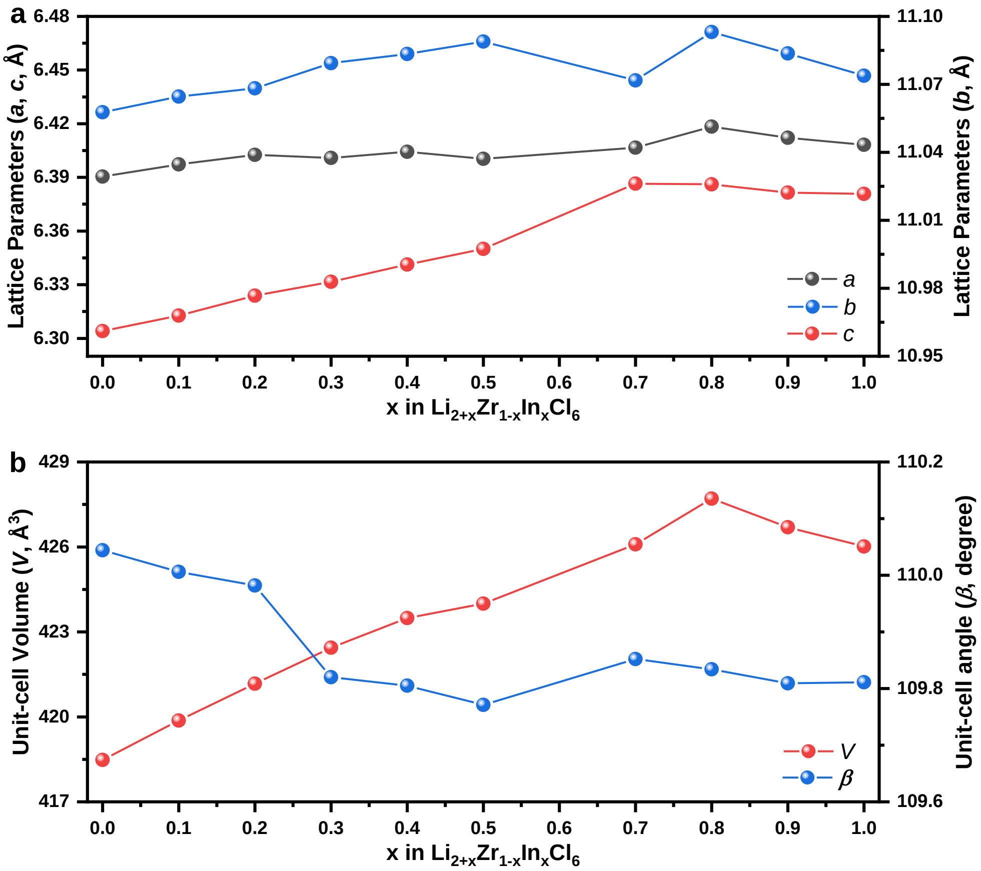

Figure S4. The lattice parameter of hc-$Li_{2+x}Zr_{1-x}In_xCl_6$ ($0 \leq x \leq 1$).

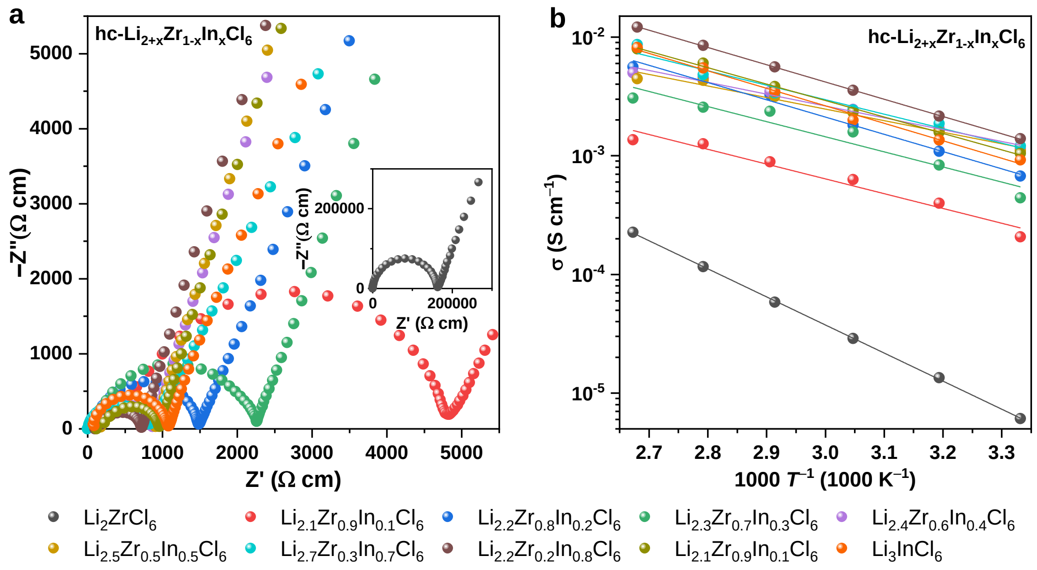

Figure S5. Nyquist (a) and Arrhenius (b) plots of the hc-$Li_{2+x}Zr_{1-x}In_xCl_6$ ($0 \leq x \leq 1$) materials.

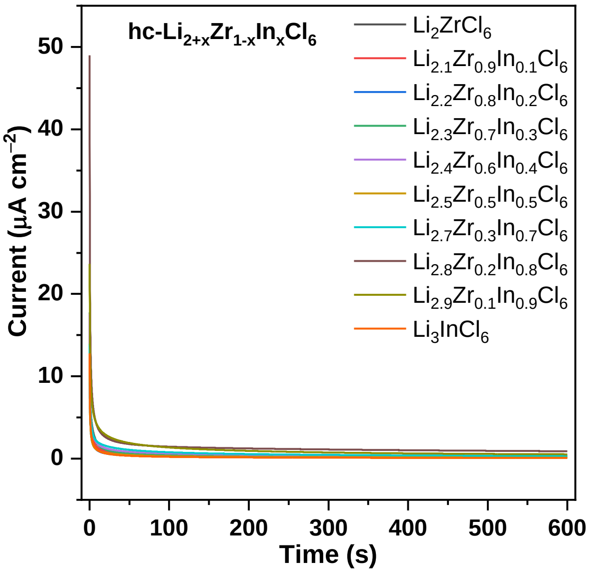

Figure S6. The transient current behaviour under an applied 1.0 V DC bias on the hc-$Li_{2+x}Zr_{1-x}In_xCl_6$ ($0 \leq x \leq 1$) pellets with stainless-steel electrodes.

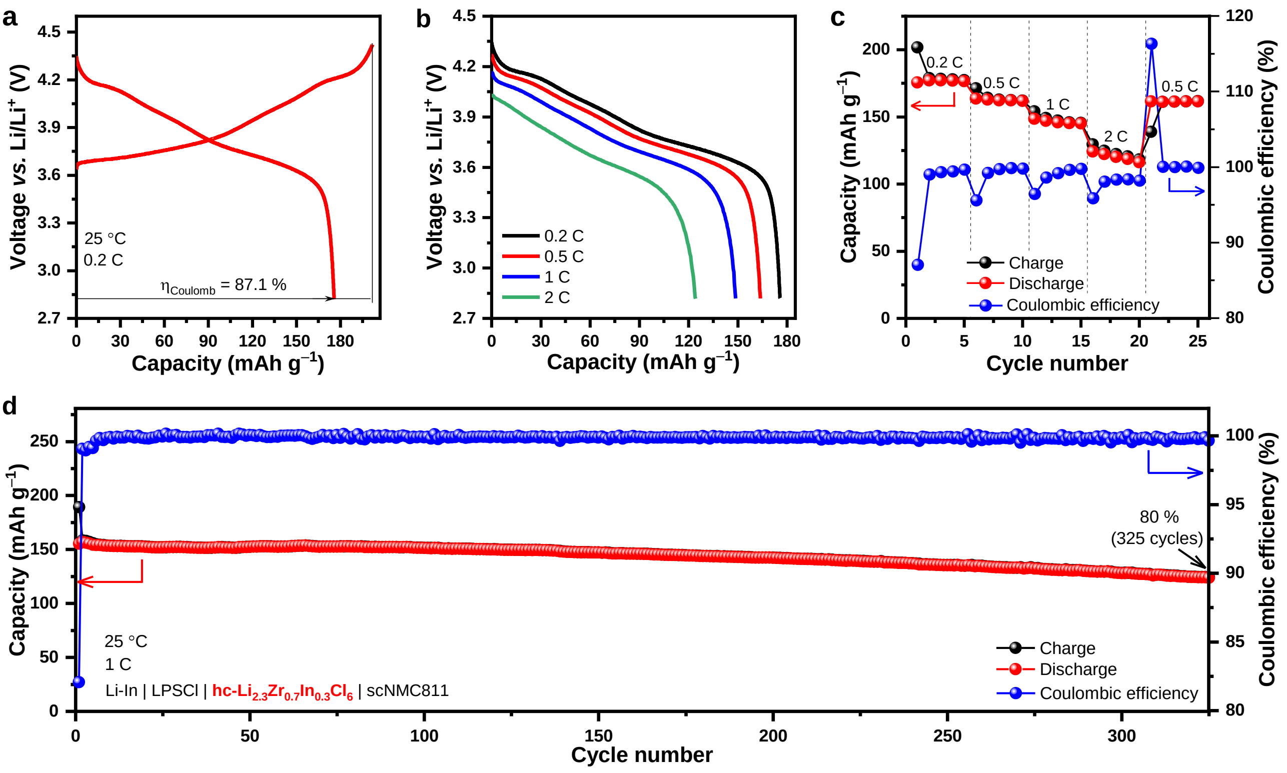

Figure S7. Electrochemical performance of the Li-In | LPSCl | hc-$Li_{2.3}Zr_{0.7}In_{0.3}Cl_6$ | scNMC811 cell. (a) The initial charge/discharge curves under 0.2 C at 25 °C. (b, c) Rate capability under 0.2, 0.5, 1, and 2 C at 25 °C. (d) Long-term cycling performance under 1 C at 25 °C.

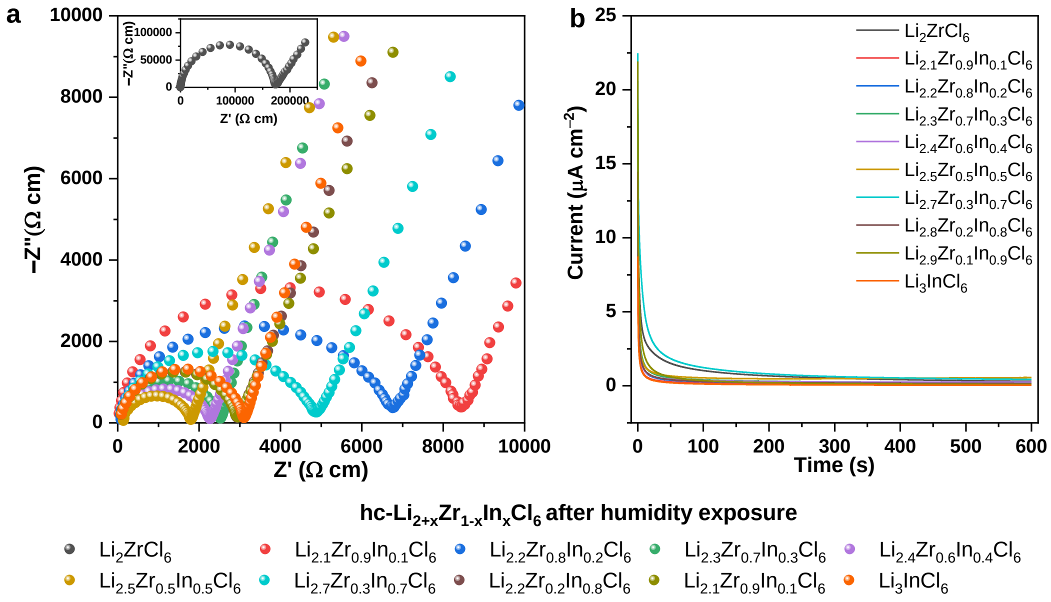

Figure S8. (a) Nyquist plots of the hc-$Li_{2+x}Zr_{1-x}In_xCl_6$ ($0 \leq x \leq 1$) after 24 hours of exposure to 5% relative humidity. (b) The transient current behaviour under an applied 1.0 V DC bias on the hc-$Li_{2+x}Zr_{1-x}In_xCl_6$ ($0 \leq x \leq 1$) after 24 hours of exposure to 5% relative humidity pellets with stainless-steel electrodes.

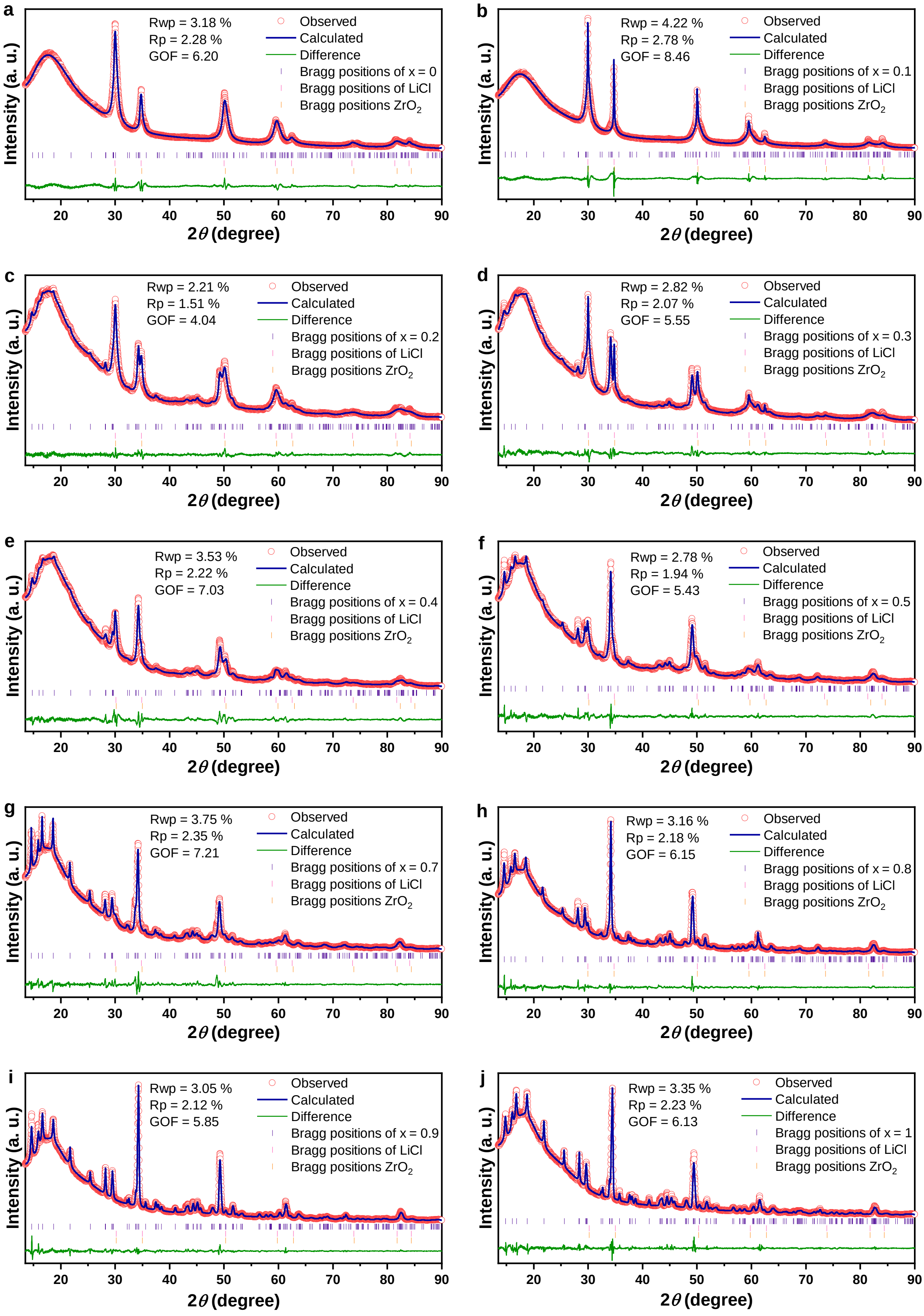

Figure S9. (a–j) Refined XRD patterns of the reheated-$Li_{2+x}Zr_{1-x}In_xCl_6$ ($0 \leq x \leq 1$).

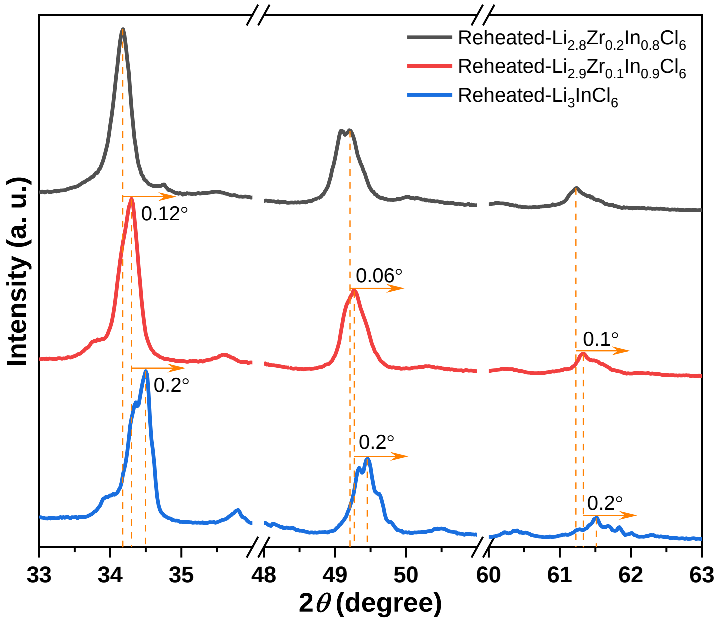

Figure S10. XRD patterns of reheated-$Li_{2.8}Zr_{0.2}In_{0.8}Cl_6$, reheated-$Li_{2.9}Zr_{0.1}In_{0.9}Cl_6$ and reheated-Li_3InCl_6.

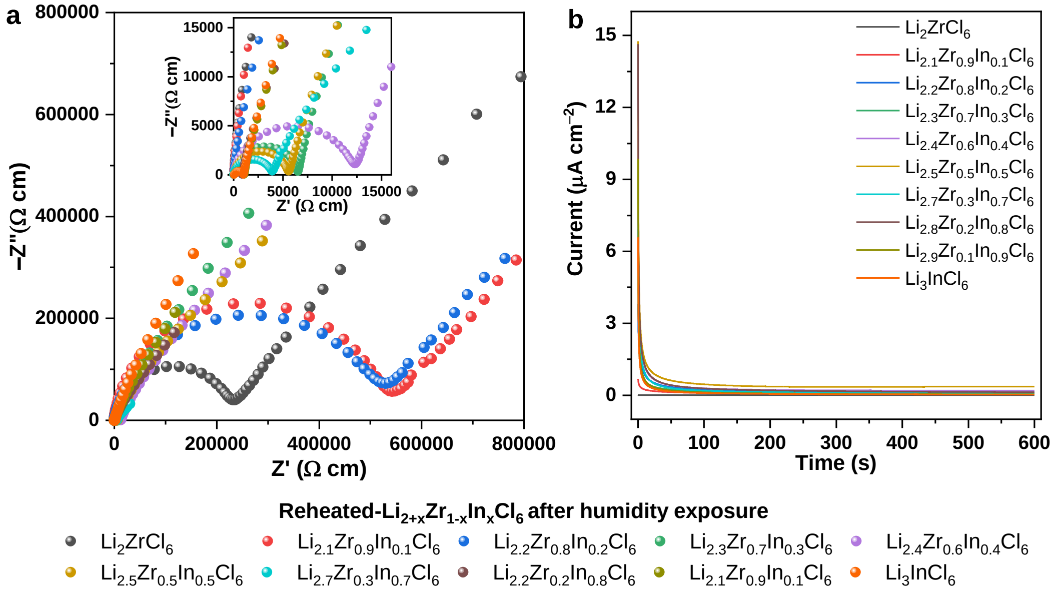

Figure S11. (a) Nyquist plots of the reheated-$Li_{2+x}Zr_{1-x}In_xCl_6$ ($0 \leq x \leq 1$) after 24 hours of exposure to 5% relative humidity. (b) The transient current behaviour under an applied 1.0 V DC bias on the reheated-$Li_{2+x}Zr_{1-x}In_xCl_6$ ($0 \leq x \leq 1$) after 24 hours of exposure to 5% relative humidity pellets with stainless-steel electrodes.

Table S1. The ionic conductivities, electronic conductivities and activation energy of lc-$Li_{2+x}Zr_{1-x}In_xCl_6$ ($0 \leq x \leq 1$).

Samples	Ionic conductivities (S cm^{-1})	Electronic conductivities (S cm^{-1})	Activation energy (eV)
Li_2ZrCl_6	8.01×10^{-4}	2.76×10^{-8}	0.35
$Li_{2.1}Zr_{0.9}In_{0.1}Cl_6$	6.96×10^{-4}	3.09×10^{-8}	0.35
$Li_{2.2}Zr_{0.8}In_{0.2}Cl_6$	6.82×10^{-4}	6.60×10^{-8}	0.35
$Li_{2.3}Zr_{0.7}In_{0.3}Cl_6$	5.43×10^{-4}	2.90×10^{-8}	0.37
$Li_{2.4}Zr_{0.6}In_{0.4}Cl_6$	4.13×10^{-4}	1.58×10^{-8}	0.36
$Li_{2.5}Zr_{0.5}In_{0.5}Cl_6$	3.65×10^{-4}	7.27×10^{-8}	0.38
$Li_{2.7}Zr_{0.3}In_{0.7}Cl_6$	2.16×10^{-4}	3.60×10^{-9}	0.41
$Li_{2.8}Zr_{0.2}In_{0.8}Cl_6$	2.96×10^{-4}	5.04×10^{-8}	0.42
$Li_{2.9}Zr_{0.1}In_{0.9}Cl_6$	4.56×10^{-4}	8.02×10^{-8}	0.38
Li_3InCl_6	4.41×10^{-4}	1.16×10^{-8}	0.39

Table S2. Rietveld refinement result from the XRD data of the hc-Li_2ZrCl_6. The space group is *C2/m*. The refined lattice parameters are a = 6.3905 (25) Å, b = 11.0578 (7) Å, c = 6.3040 (23) Å and β = 110.044 (6)°. The unit-cell volume is 418.49 (5) Å^3.

Atoms	x	y	z	Occ.	site	Sym.	U_{iso} (Å^2)
Li1	0	0.17 (13)	1/2	0.5	4*h*	2	0.60 (10)
Li2	1/2	0	1/2	1	2*d*	2/m	0.60 (10)
Zr1	0	0	0	0.746 (27)	2*a*	2/m	0.0213 (10)
Zr2	0	1/3	0	0.126 (8)	4*g*	2	0.0213 (10)
Cl1	0.2474 (11)	0.1643 (4)	0.2352 (6)	1	8*j*	1	0.0289 (10)
Cl2	0.2552 (13)	0	0.7723 (10)	1	4*i*	m	0.0289 (10)

Table S3. Rietveld refinement result from the XRD data of the hc-$Li_{2.1}Zr_{0.9}In_{0.1}Cl_6$. The space group is *C2/m*. The refined lattice parameters are a = 6.3973 (19) Å, b = 11.0644 (5) Å, c = 6.3127 (16) Å and β = 110.006 (5)°. The unit-cell volume is 419.87 (5) $Å^3$.

Atoms	x	y	z	Occ.	site	Sym.	U_{iso} ($Å^2$)
Li1	0	0.17 (13)	1/2	0.55	4*h*	2	1.6 (3)
Li2	1/2	0	1/2	1	2*d*	2/m	1.6 (3)
Zr1	0	0	0	0.709 (9)	2*a*	2/m	0.0157 (22)
Zr2	0	1/3	0	0.095 (3)	4*g*	2	0.0157 (22)
In1	0	0	0	0.028 (22)	2*a*	2/m	0.201 (12)
In2	0	1/3	0	0.035 (8)	4*g*	2	0.201 (12)
Cl1	0.2412 (10)	0.1620 (5)	0.2371 (6)	1	8*j*	1	0.0358 (12)
Cl2	0.2488 (13)	0	0.7659 (11)	1	4*i*	m	0.0358 (12)

Table S4. Rietveld refinement result from the XRD data of the hc-$Li_{2.2}Zr_{0.8}In_{0.2}Cl_6$. The space group is *C2/m*. The refined lattice parameters are a = 6.4026 (17) Å, b = 11.0684 (6) Å, c = 6.3239 (15) Å and β = 109.982 (4)°. The unit-cell volume is 421.17 (4) Å^3.

Atoms	x	y	z	Occ.	site	Sym.	U_{iso} (Å^2)
Li1	0	0.17 (3)	1/2	0.6	4*h*	2	0.54 (5)
Li2	1/2	0	1/2	1	2*d*	2/m	0.54 (5)
Zr1	0	0	0	0.624 (8)	2*a*	2/m	0.025 (5)
Zr2	0	1/3	0	0.087 (5)	4*g*	2	0.025 (5)
In1	0	0	0	0.133 (16)	2*a*	2/m	0.008 (11)
In2	0	1/3	0	0.033 (4)	4*g*	2	0.008 (11)
Cl1	0.2415 (9)	0.1629 (4)	0.2391 (5)	1	8*j*	1	0.0349 (9)
Cl2	0.2484 (10)	0	0.7663 (8)	1	4*i*	m	0.0349 (9)

Table S5. Rietveld refinement result from the XRD data of the hc-$Li_{2.3}Zr_{0.7}In_{0.3}Cl_6$. The space group is *C2/m*. The refined lattice parameters are a = 6.4011 (22) Å, b = 11.0800 (8) Å, c = 6.3321 (21) Å and β = 109.820 (6)°. The unit-cell volume is 422.50 (4) Å^3.

Atoms	x	y	z	Occ.	site	Sym.	U_{iso} (Å^2)
Li1	0	0.1683 (27)	1/2	0.65	4*h*	2	0.49 (6)
Li2	1/2	0	1/2	1	2*d*	2/m	0.49 (6)
Zr1	0	0	0	0.392 (8)	2*a*	2/m	0.008 (6)
Zr2	0	1/3	0	0.153 (5)	4*g*	2	0.008 (6)
In1	0	0	0	0.234 (8)	2*a*	2/m	0.047 (16)
In2	0	1/3	0	0.032 (5)	4*g*	2	0.047 (16)
Cl1	0.2426 (20)	0.1626 (5)	0.2378 (7)	1	8*j*	1	0.0387 (17)
Cl2	0.2508 (22)	0	0.7662 (13)	1	4*i*	m	0.0387 (17)

Table S6. Rietveld refinement result from the XRD data of the hc-$Li_{2.4}Zr_{0.6}In_{0.4}Cl_6$. The space group is *C2/m*. The refined lattice parameters are a = 6.4041 (22) Å, b = 11.0836 (7) Å, c = 6.3412 (20) Å and β = 109.805 (5)°. The unit-cell volume is 423.48 (5) Å^3.

Atoms	x	y	z	Occ.	site	Sym.	U_{iso} (Å^2)
Li1	0	0.1688 (3)	1/2	0.7	4*h*	2	0.222 (3)
Li2	1/2	0	1/2	1	2*d*	2/m	0.222 (3)
Zr1	0	0	0	0.307 (4)	2*a*	2/m	0.013 (15)
Zr2	0	1/3	0	0.146 (3)	4*g*	2	0.013 (15)
In1	0	0	0	0.330 (9)	2*a*	2/m	0.034 (22)
In2	0	1/3	0	0.034 (5)	4*g*	2	0.034 (22)
Cl1	0.2424 (16)	0.1616 (4)	0.2339 (6)	1	8*j*	1	0.028 (12)
Cl2	0.2486 (18)	0	0.7609 (11)	1	4*i*	m	0.028 (12)

Table S7. Rietveld refinement result from the XRD data of the hc-$Li_{2.5}Zr_{0.5}In_{0.5}Cl_6$. The space group is *C2/m*. The refined lattice parameters are a = 6.400 (10) Å, b = 11.0918 (26) Å, c = 6.350 (9) Å and β = 109.771 (27)°. The unit-cell volume is 424.17 (13) Å^3.

Atoms	x	y	z	Occ.	site	Sym.	U_{iso} (Å^2)
Li1	0	0.168 (6)	1/2	0.75	4*h*	2	0.172 (31)
Li2	1/2	0	1/2	1	2*d*	2/m	0.172 (31)
Zr1	0	0	0	0.347 (6)	2*a*	2/m	0.02 (8)
Zr2	0	1/3	0	0.076 (15)	4*g*	2	0.02 (8)
In1	0	0	0	0.350 (7)	2*a*	2/m	0.02 (7)
In2	0	1/3	0	0.074 (9)	4*g*	2	0.02 (7)
Cl1	0.2244 (13)	0.1606 (8)	0.2425 (12)	1	8*j*	1	0.0334 (14)
Cl2	0.2611 (19)	0	0.7481 (20)	1	4*i*	m	0.0334 (14)

Table S8. Rietveld refinement result from the XRD data of the hc-$Li_{2.7}Zr_{0.3}In_{0.7}Cl_6$. The space group is *C2/m*. The refined lattice parameters are a = 6.4073 (29) Å, b = 11.0707 (10) Å, c = 6.3873 (29) Å and β = 109.852 (7)°. The unit-cell volume is 426.15 (7) Å^3.

Atoms	x	y	z	Occ.	site	Sym.	U_{iso} (Å^2)
Li1	0	0.1683 (4)	1/2	0.85	4*h*	2	0.054 (15)
Li2	1/2	0	1/2	1	2*d*	2/m	0.054 (15)
Zr1	0	0	0	0.176 (21)	2*a*	2/m	0.094 (19)
Zr2	0	1/3	0	0.061 (8)	4*g*	2	0.094 (19)
In1	0	0	0	0.609 (3)	2*a*	2/m	0.0038 (18)
In2	0	1/3	0	0.045 (3)	4*g*	2	0.0038 (18)
Cl1	0.2616 (9)	0.1599 (4)	0.2499 (8)	1	8*j*	1	0.0529 (22)
Cl2	0.2279 (10)	0	0.7614 (11)	1	4*i*	m	0.0529 (22)

Table S9. Rietveld refinement result from the XRD data of the hc-$Li_{2.8}Zr_{0.2}In_{0.8}Cl_6$. The space group is *C2/m*. The refined lattice parameters are a = 6.4182 (24) Å, b = 11.0938 (7) Å, c = 6.3856 (23) Å and β = 109.834 (6)°. The unit-cell volume is 427.70 (5) Å^3.

Atoms	x	y	z	Occ.	site	Sym.	U_{iso} (Å^2)
Li1	0	0.17 (5)	1/2	0.9	4*h*	2	0.208 (15)
Li2	1/2	0	1/2	1	2*d*	2/m	0.208 (15)
Zr1	0	0	0	0.152 (10)	2*a*	2/m	0.01 (4)
Zr2	0	1/3	0	0.023 (9)	4*g*	2	0.01 (4)
In1	0	0	0	0.609 (7)	2*a*	2/m	0.021 (8)
In2	0	1/3	0	0.095 (13)	4*g*	2	0.021 (8)
Cl1	0.2432 (10)	0.1620 (4)	0.2391 (5)	1	8*j*	1	0.0319 (7)
Cl2	0.2448 (12)	0	0.7633 (7)	1	4*i*	m	0.0319 (7)

Table S10. Rietveld refinement result from the XRD data of the hc-$Li_{2.9}Zr_{0.1}In_{0.9}Cl_6$. The space group is *C2/m*. The refined lattice parameters are a = 6.4122 (11) Å, b = 11.0839 (3) Å, c = 6.3816 (11) Å and β = 109.8092 (27)°. The unit-cell volume is 426.707 (25) Å^3.

Atoms	x	y	z	Occ.	site	Sym.	U_{iso} (Å^2)
Li1	0	0.1723 (22)	1/2	0.95	4*h*	2	0.967 (6)
Li2	1/2	0	1/2	1	2*d*	2/m	0.967 (6)
Zr1	0	0	0	0.048 (25)	2*a*	2/m	0.35 (6)
Zr2	0	1/3	0	0.025 (9)	4*g*	2	0.35 (6)
In1	0	0	0	0.717 (11)	2*a*	2/m	0.0052 (7)
In2	0	1/3	0	0.091 (4)	4*g*	2	0.0052 (7)
Cl1	0.2411 (7)	0.1639 (28)	0.2403 (4)	1	8*j*	1	0.0473 (11)
Cl2	0.2420 (8)	0	0.7668 (7)	1	4*i*	m	0.0473 (11)

Table S11. Rietveld refinement result from the XRD data of the hc-Li_3InCl_6. The space group is *C2/m*. The refined lattice parameters are a = 6.4082 (8) Å, b = 11.0745 (25) Å, c = 6.3812 (7) Å and β = 109.8111 (19)°. The unit-cell volume is 426.056 (18) Å^3.

Atoms	x	y	z	Occ.	site	Sym.	U_{iso} (Å^2)
Li1	0	0.1683	1/2	1	4*h*	2	0.40 (3)
Li2	1/2	0	1/2	1	2*d*	2/m	0.40 (3)
In1	0	0	0	0.810 (27)	2*a*	2/m	0.0237 (6)
In2	0	1/3	0	0.094 (8)	4*g*	2	0.0237 (6)
Cl1	0.2425 (6)	0.1653 (3)	0.2403 (4)	1	8*j*	1	0.0246 (6)
Cl2	0.2439 (8)	0	0.7636 (6)	1	4*i*	m	0.0246 (6)

Table S12. The ionic conductivities, electronic conductivities and activation energy of hc-$Li_{2+x}Zr_{1-x}In_xCl_6$ ($0 \le x \le 1$).

Samples	Ionic conductivities (S cm^{-1})	Electronic conductivities (S cm^{-1})	Activation energy (eV)
Li_2ZrCl_6	6.11×10^{-6}	1.89×10^{-8}	0.50
$Li_{2.1}Zr_{0.9}In_{0.1}Cl_6$	2.08×10^{-4}	1.83×10^{-8}	0.31
$Li_{2.2}Zr_{0.8}In_{0.2}Cl_6$	6.73×10^{-4}	1.67×10^{-8}	0.32
$Li_{2.3}Zr_{0.7}In_{0.3}Cl_6$	9.69×10^{-4}	3.35×10^{-8}	0.28
$Li_{2.4}Zr_{0.6}In_{0.4}Cl_6$	1.15×10^{-3}	3.27×10^{-8}	0.25
$Li_{2.5}Zr_{0.5}In_{0.5}Cl_6$	1.10×10^{-3}	1.67×10^{-8}	0.24
$Li_{2.7}Zr_{0.3}In_{0.7}Cl_6$	1.21×10^{-3}	2.99×10^{-8}	0.27
$Li_{2.8}Zr_{0.2}In_{0.8}Cl_6$	1.39×10^{-3}	1.11×10^{-7}	0.32
$Li_{2.9}Zr_{0.1}In_{0.9}Cl_6$	1.04×10^{-3}	5.14×10^{-8}	0.30
Li_3InCl_6	9.23×10^{-4}	8.35×10^{-9}	0.32

Table S13. The ionic conductivities and electronic conductivities of hc-$Li_{2+x}Zr_{1-x}In_xCl_6$ ($0 \leq x \leq 1$) after 24 hours of exposure to 5% relative humidity.

Samples	Ionic conductivities (S cm^{-1})	Electronic conductivities (S cm^{-1})
Li_2ZrCl_6	5.77×10^{-6}	5.07×10^{-8}
$Li_{2.1}Zr_{0.9}In_{0.1}Cl_6$	1.18×10^{-4}	1.08×10^{-8}
$Li_{2.2}Zr_{0.8}In_{0.2}Cl_6$	1.48×10^{-4}	2.04×10^{-8}
$Li_{2.3}Zr_{0.7}In_{0.3}Cl_6$	3.95×10^{-4}	2.15×10^{-8}
$Li_{2.4}Zr_{0.6}In_{0.4}Cl_6$	4.40×10^{-4}	2.92×10^{-8}
$Li_{2.5}Zr_{0.5}In_{0.5}Cl_6$	5.53×10^{-4}	6.55×10^{-8}
$Li_{2.7}Zr_{0.3}In_{0.7}Cl_6$	2.04×10^{-4}	4.91×10^{-8}
$Li_{2.8}Zr_{0.2}In_{0.8}Cl_6$	3.40×10^{-4}	1.87×10^{-8}
$Li_{2.9}Zr_{0.1}In_{0.9}Cl_6$	3.38×10^{-4}	1.10×10^{-8}
Li_3InCl_6	3.22×10^{-4}	4.29×10^{-9}

Table S14. Composition of the reheated-$Li_{2+x}Zr_{1-x}In_xCl_6$ ($0 \le x \le 1$).

Samples	Reheated-$Li_{2+x}Zr_{1-x}In_xCl_6$ ($0 \le x \le 1$) / Phase fraction, %	LiCl / Phase fraction, %	ZrO_2 / Phase fraction, %
x = 0	0	53.59	46.41
x = 0.1	0	65.47	34.53
x = 0.2	11.58	62.62	25.80
x = 0.3	35.55	35.31	29.14
x = 0.4	67.12	21.8	11.08
x = 0.5	68.44	20.19	11.37
x = 0.7	89.93	5.98	4.09
x = 0.8	96.96	1.92	1.12
x = 0.9	100	0	0
x = 1.0	100	0	0

Table S15. Rietveld refinement result from the room-temperature XRD data of the reheated-$Li_{2.8}Zr_{0.2}In_{0.8}Cl_6$. The space group is *C2/m*. The refined lattice parameters are *a* = 6.417(3) Å, *b* = 11.0828(16) Å, *c* = 6.382(3) Å, $\alpha = \gamma = 90$ °,and β = 109.771(7)°, unit-cell volume = 427.137(17) Å^3.

Atoms	x	y	z	Occ.	site	Sym.	U_{iso} (Å^2)
Li1	0	0.164(7)	1/2	0.9	4*h*	2	0.255(24)
Li2	1/2	0	1/2	1	2*d*	2/m	0.255(24)
Zr1	0	0	0	0.15(4)	2*a*	2/m	0.01(11)
Zr2	0	1/3	0	0.025(19)	4*g*	2	0.01(11)
In1	0	0	0	0.609(3)	2*a*	2/m	0.011(21)
In2	0	1/3	0	0.096(15)	4*g*	2	0.011(21)
Cl1	0.2432(10)	0.1624(4)	0.2397(5)	1	8*j*	1	0.0328(11)
Cl2	0.2434(11)	0	0.7620(8)	1	4*i*	m	0.0328(11)

Table S16. Rietveld refinement result from the room-temperature XRD data of the reheated-$Li_{2.9}Zr_{0.1}In_{0.9}Cl_6$. The space group is *C2/m*. The refined lattice parameters are a = 6.411(3) Å, b = 11.0825(15) Å, c = 6.3824(31) Å, $\alpha = \gamma = 90$ °,and β= 109.806(7)°, unit-cell volume = 426.66(17) Å^3.

Atoms	x	y	z	Occ.	site	Sym.	U_{iso} (Å^2)
Li1	0	0.160(8)	1/2	0.95	4*h*	2	0.67(10)
Li2	1/2	0	1/2	1	2*d*	2/m	0.67(10)
Zr1	0	0	0	0.046(4)	2*a*	2/m	0.15(6)
Zr2	0	1/3	0	0.0272(19)	4*g*	2	0.15(6)
In1	0	0	0	0.7338(19)	2*a*	2/m	0.0057(10)
In2	0	1/3	0	0.0831(10)	4*g*	2	0.0057(10)
Cl1	0.2398(7)	0.16254(28)	0.2407(4)	1	8*j*	1	0.0426(11)
Cl2	0.2418(8)	0	0.7639(6)	1	4*i*	m	0.0426(11)

Table S17. Rietveld refinement result from the room-temperature XRD data of the reheated-Li_3InCl_6. The space group is *C2/m*. The refined lattice parameters are a = 6.4080(20) Å, b = 11.0747(9) Å, c = 6.3810(20) Å, $\alpha = \gamma = 90$ °,and β = 109.809(5)°, unit-cell volume = 426.05(10) Å^3.

Atoms	x	y	z	Occ.	site	Sym.	U_{iso} (Å^2)
Li1	0	0.165(9)	1/2	1	4*h*	2	0.45(4)
Li2	1/2	0	1/2	1	2*d*	2/m	0.45(4)
In1	0	0	0	0.8615(24)	2*a*	2/m	0.0024(13)
In2	0	1/3	0	0.0692(12)	4*g*	2	0.0024(13)
Cl1	0.2418(8)	0.1652(4)	0.2435(6)	1	8*j*	1	0.0367(13)
Cl2	0.2440(11)	0	0.7654(9)	1	4*i*	m	0.0367(13)

Table S18. The ionic conductivities and electronic conductivities of reheated-$Li_{2+x}Zr_{1-x}In_xCl_6$ ($0 \leq x \leq 1$) after 24 hours of exposure to 5% relative humidity.

Samples	Ionic conductivities (S cm^{-1})	Electronic conductivities (S cm^{-1})
Li_2ZrCl_6	4.25×10^{-6}	7.93×10^{-10}
$Li_{2.1}Zr_{0.9}In_{0.1}Cl_6$	1.84×10^{-6}	6.15×10^{-9}
$Li_{2.2}Zr_{0.8}In_{0.2}Cl_6$	1.89×10^{-6}	1.42×10^{-8}
$Li_{2.3}Zr_{0.7}In_{0.3}Cl_6$	1.52×10^{-4}	9.84×10^{-9}
$Li_{2.4}Zr_{0.6}In_{0.4}Cl_6$	8.10×10^{-5}	2.16×10^{-8}
$Li_{2.5}Zr_{0.5}In_{0.5}Cl_6$	1.78×10^{-4}	4.21×10^{-8}
$Li_{2.7}Zr_{0.3}In_{0.7}Cl_6$	2.53×10^{-4}	8.18×10^{-9}
$Li_{2.8}Zr_{0.2}In_{0.8}Cl_6$	7.49×10^{-4}	1.43×10^{-8}
$Li_{2.9}Zr_{0.1}In_{0.9}Cl_6$	8.71×10^{-4}	4.47×10^{-9}
Li_3InCl_6	1.07×10^{-3}	3.03×10^{-9}
